# Category characteristics of high school students’ sense of hope and its relationship with mental health

**DOI:** 10.3389/fpsyg.2023.1140480

**Published:** 2023-02-20

**Authors:** Rui Wang, Siyu Di, Yajing Sun, Yafei Liu, Chao Ma

**Affiliations:** ^1^Normal College, Shihezi University, Shihezi, China; ^2^Center of Application of Psychological Research, Shihezi University, Shihezi, China

**Keywords:** high school students, sense of hope, mental health, latent profile analysis, category characteristics

## Abstract

**Purpose:**

This study used the latent profile analysis technique to investigate the latent categories of high school students’ sense of hope and their relationship with mental health.

**Methods:**

A total of 1,513 high school students from six middle schools in China was tested using the Adult Dispositional Hope Scale and the Symptom Checklist 90. Analysis of variance was used to explore the relationship between latent categories of sense of hope and mental health.

**Results:**

High school students’ sense of hope scores are negatively associated with mental health scores. The high school students’ sense of hope could be divided into three latent categories: negative sense of hope group, moderate sense of hope group and positive sense of hope group. The differences in scores on each dimension of mental health among high school students with different latent categories of sense of hope were statistically significant. The positive sense of hope group had lower scores on the dimensions of somatization, compulsive symptoms, interpersonal sensitivity, depression, anxiety, hostility, terror, paranoia, and psychosis than the negative sense of hope group and the moderate sense of hope group.

**Conclusion:**

There are three latent categories of high school students’ sense of hope, and the sense of hope category is closely related to mental health. Based on the different categories of high school students’ sense of hope, the program of mental health education can be reasonably selected to create a positive general environment for mental health education and ultimately enhance the mental health of high school students.

## 1. Introduction

Hope is the desire for something attainable or strongly believed to be attainable and the resulting positive affective and behavioral tendencies. Miller and Agency point out from an essential and etymological perspective that a sense of hope is the expectation and imagination of something promising or a state of fulfillment, while exhibiting the thinking and emotions of refining oneself and unleashing one’s potential to achieve this goal (Miller and Powers, 1988). As a positive psychological quality that points to the future, a sense of hope has a powerful intrapsychic drive and goal orientation. Through the interaction of pathway thinking and agency thinking, the sense of hope motivates individuals to generate positive emotions and feelings in the process of achieving desired goals, thereby positively advancing their mental health development (Xiao Qian and Hua, 2013). When reviewing the research related to sense of hope, it is found that there are still some deficiencies in the current research. Firstly, previous studies have mostly adopted a variable-centered approach to investigate the effects of hope on mental health, ignoring the holistic nature of individual development, which means that there may be differences in the types of hope among individuals. Secondly, different cultural backgrounds or social environments may lead to differences in the types of the individual sense of hope. Therefore, it is inevitable to examine the types of sense of hope based on the current Chinese socio-cultural system (Zhang et al., 2022). High school is a critical period for students’ physical and mental development, which is also accompanied by diverse psychological problems (Yu and Yu, 2022). Therefore, this study intends to adopt an individual-centered approach to explore different types of sense of hope and their differences in the dimensions of sense of hope among current high school students, and to further explore the mental health status of high school students with different types of sense of hope in order to provide implications for improving the mental health of high school students.

Through the literature, the concept of sense of hope has been divided into two schools of thought, namely, the emotional view of hope and the cognitive view of hope. Erikson hypothesizes that hope is a factor in overall cognitive development, and he argues that strong desire arises from internal conflict during the individual’s developmental period and is more pronounced in early childhood. Gottschalk views hope as a persistent, strong obsession that individuals develop to achieve a goal, which motivates them to perform continuous actions around the goal. Breznik states that hope belongs to the cognitive category, and he considers hope as a cognitive state and psychological response of continuous reaction. Mower argues from an activist perspective that hope is an emotion similar to a stimulus reinforcer. In the stimulus–response model, when a subject perceives a stimulus accompanied by a good outcome, the frequency of such stimulus will be increased to obtain a pleasurable response, which means that the subject has the emotional experience of hope. Verill disputes this, stating that hope is an affective experience around a goal, which naturally arises when the individual’s goal is manageable, normative, and meaningful (Yanxiang, 2021). Snyder’s theory of hope, which is widely accepted in psychology, is based on previous research and considers hope as a positively motivated state of mind that includes both agency thinking and pathway thinking. Pathway thinking refers to the measures that individuals take to accomplish their goals. Agency thinking refers to an individual’s ability to make plans to accomplish a goal (Snyder, 2002). Recently, some researchers have pointed out that agency thinking and pathway thinking are both functionally related and different. Both pathway thinking and agency thinking have significant predictive effects on positive emotions. However, when predicting negative emotions, agency thinking alleviates individuals’ negative emotions, while pathway thinking deepens them (Peng et al., 2020). In recent years, researchers have commonly adopted these two dimensions to develop sense of hope questionnaires, such as the Trait Sense of Hope Scale and the State Sense of Hope Scale for adults, as well as the Children’s Sense of Hope Scale (Zhang et al., 2022). Therefore, this study aims to explore the types of sense of hope of high school students based on a two-factor theoretical framework.

Numerous studies have found a positive relationship between sense of hope and individuals’ psychological well-being, but with slight differences in research perspectives. Firstly, a sense of hope is significantly and negatively related to suicidal ideation (O'Keefe and Wingate, 2013), which dissipates negative emotions that affect individuals’ physical and mental health (Rock et al., 2014), and achieve the purpose of eliminating individuals’ suicidal behavioral ideation. Individuals with high levels of hope have a higher sense of self-efficacy and positive expectations when faced with life stress. Their attention shifts from focusing on the negative outcome of the event to thinking about solutions to the problem. They are more inclined to take action to get out of the current dilemma, leading to less suicidal ideation (Yuan Qunming, 2021). The opposite is true for individuals with a low sense of hope. They lack positive attitudes in the face of stress and distress and are prone to pessimism, negative reactions of disappointment and negative attributional thinking. These reinforce the negative effects of stress and lead to higher levels of suicidal ideation (Xiang Guangcan et al., 2020). This has been confirmed in studies with adolescent populations. A sense of hope can alleviate the insecurities and discontentment that foster children experience as they grow up. A sense of hope also had a moderating effect on students who experienced childhood abuse that led to suicidal behavior in adulthood. In addition, the sense of hope mitigates the negative effects of violent acts and traumatic events on adolescents (Yang et al., 2013).

Conversely, as a positive psychological motivator, a sense of hope promotes positive psychological qualities in high school students and significantly improves their mental health (Chen et al., 2021). Numerous studies have shown that individuals with a high sense of hope have a clear sense of self-perception and meaning in life (Snyder, 2002). They have an attitude of gratitude for the current bright life and a positive attitude toward the people and things in their lives. When failures occur, individuals with a sense of hope are more optimistic and confident, attribute the failure to a lack of effort and a deviation in strategy rather than to a deficiency in their own abilities, and mobilize resources to achieve their goals as much as possible (McCullough, 2002). That is, individuals with a high sense of hope exhibit higher self-esteem, greater self-efficacy, and more positive behavioral performance. In contrast, individuals with a low sense of hope are more inclined to dwell on negative emotions such as depression and are prone to more outward behavioral problems (Xiao Qian and Hua, 2013). In addition, recent studies have found that a sense of hope can affect an individual’s physical and mental health through two mechanisms: buffering and catalysis. The effects of these two mechanisms are significantly enhanced when the individual’s psychological state deteriorates further. Firstly, when an individual is in an unhealthy psychological state, a high sense of hope has a healing effect on the individual’s mental and physical health, thereby buffering suicidal ideation. Secondly, under the same circumstances, individuals with a lower sense of hope tend to amplify the negative effects of negative emotions and thereby catalyze their suicidal behavior. Therefore, the effect of sense of hope on an individual’s mental health is not simply a matter of alleviating or eliminating the adverse effects, but has different mechanisms of action based on different levels (Yanxiang, 2021).

Latent profile analysis (LPA) is an emerging individual-centered statistical analysis method that elucidates the association between external continuous variables by analyzing latent categories of variables to achieve relative local independence of exogenous variables. Compared with the traditional mean partitioning and cluster analysis, LPA classification is more accurate and objective, and the model fit is better (Magidson and Vermunt, 2002). Currently, latent profile analysis is widely used in the fields of education, psychology, and sociology in China, such as Big Five personality, organizational commitment, psychological contract, and work motivation. In addition, latent profile analysis is an extension of the latent category model for continuous exogenous variables, which can distinguish both qualitative and quantitative differences among individuals (Tennen et al., 2002).

In summary, this study intends to adopt a latent profile analysis to investigate the types of sense of hope and its relationship with mental health status among high school students. There is empirical research support for either the positive or negative view of both dimensions of sense of hope on adolescent psychological well-being, thus further in-depth exploration of the relationship is warranted. In addition, based on previous studies that have examined the types of hope using a two-factor theoretical framework, although the classification of the types of sense of hope is controversial, the studies agree that high sense of hope high school students have better psychological health than low sense of hope high school students. Therefore, this study concluded that at least two types of hope can be classified, namely a high sense of hope and low sense of hope, and hypothesized that high school students with low sense of hope have lower levels of mental health.

## 2. Materials and methods

### 2.1. Participants

In this study, a whole group sampling method was used to select students from six high schools in China as the subjects. The age range of the subjects was 16–18 years. A total of 1,676 questionnaires were distributed, 163 questionnaires were excluded from the omissions and regular responses, and finally 1,513 valid questionnaires were obtained, with an efficiency rate of 90.27%. Among the valid questionnaires, 718 were male students with a mean age of 17.94 years (SD = 1.32) and 795 were female students with a mean age of 17.77 years (SD = 1.29). There were 623, 486, and 404 students in the first, second, and third year of high school, respectively.

### 2.2. Materials

#### 2.2.1. Sense of hope

Sense of hope was assessed using the Adult Dispositional Hope Scale (J, 2006). The scale consists of 12 items divided into two dimensions: path thinking (4 items) and agency thinking (4 items). Subjects answered each item using a 4-point Likert scale. The total score ranges from 8 to 32, with higher scores representing higher levels of hopeful traits. Four of the entries, 3, 5, 7, and 11, were used to divert the subjects’ attention and were not included in the total score. The Cronbach’s α of the scale in this study was 0.938.

#### 2.2.2. Mental health

The Symptom Checklist 90 (SCL-90) scale was used to measure mental health (Zhengyu, 1984). The scale has 90 items divided into 10 dimensions: somatization, compulsive symptoms, interpersonal sensitivity, depression, anxiety, hostility, terror, paranoia, psychosis, and other reasons. Subjects answered each item using a 5-point Likert scale, with higher scores indicating poorer mental health. The Cronbach’s α of the scale in this study was 0.967.

### 2.3. Statistical processing and analysis plan

The researcher contacted six schools through whole group sampling, and after communication, an online anonymous questionnaire was distributed, which took approximately 20 min to complete. The study used SPSS 26.0 for data entry and descriptive statistical analysis of relevant variables, M plus 7.4 for latent profile analysis of sense of hope and examined the relationship between different profiles of sense of hope and other variables. Specifically, the data analysis consisted of three parts. In the first part, the latent profiles of hope sense were analyzed by latent profile analysis to seek the model with the best fitting index. In the second part, one-way ANOVA was used to analyze the differences between adolescents with different types of sense of hope on the total score and each dimension of the sense of hope. In the third part, the results of the sense of hope profiles derived from the first step were used as independent variables to analyze whether the latent categories of sense of hope differed significantly on different dimensions of adolescent mental health.

## 3. Results

### 3.1. Common method bias test

To avoid common method bias, the study used the Harman one-way test for common method bias. The results showed that the variance explained by the first factor was 24.57%, which was less than the critical criterion of 40%. Therefore, there is no significant common method bias in this study.

### 3.2. Descriptive statistics and correlation analysis

The means, standard deviations, and correlation coefficients of the variables in this study are shown in Table 1. Among them, gender was significantly correlated with each factor of SCL-90, and the grade was significantly positively correlated with pathway thinking (*r* = 0.09, *p* < 0.01) and agency thinking (*r* = 0.13, *p* < 0.01). Except for the anxiety variable, pathway thinking and agency thinking were significantly and negatively correlated with each of the mental health factors.

**Table 1:** Summary of means, standard deviations and correlation coefficients for each variable.

Variable	1	2	3	4	5	6	7	8	9	10	11	12	13	14	15
1. Gender	1														
2. Only-child	0.09*	1													
3. Grade	−0.06*	−0.03	1												
4. Pathway thinking	0.03	−0.02	0.09 **	1											
5. Agency thinking	0.01	−0.02	0.10 **	0.86 **	1										
6. Somatization	−0.08 **	−0.04	−0.05	−0.07 *	−0.07 **	1									
7. Compulsive symptoms	−0.15 **	0.01	0.00	−0.17 **	−0.11 **	0.57 **	1								
8. Interpersonal sensitivity	−0.10 **	−0.02	0.02	−0.11 **	−0.07 **	0.56 **	0.75 **	1							
9. Depression	−0.14 **	−0.03	−0.03	−0.11 **	−0.08 **	0.66 **	0.74 **	0.80 **	1						
10. Anxiety	−0.12	−0.03	−0.03	−0.12	−0.09	0.67	0.72	0.75	0.79	1					
11. Hostility	−0.09 **	−0.01	−0.01	−0.08 **	−0.06 *	0.60 **	0.61 **	0.62 **	0.69 **	0.68 **	1				
12. Terror	−0.20 **	−0.03	0.02	−0.10 **	−0.05	0.55 **	0.67 **	0.71 **	0.69 **	0.72 **	0.57 **	1			
13. Paranoia	−0.06 *	−0.03	−0.02	−0.09 **	−0.07 **	0.61 **	0.66 **	0.75 **	0.75 **	0.72 **	0.65 **	0.66 **	1		
14. Psychosis	−0.07 **	−0.03	−0.02	−0.10 **	−0.08 **	0.60 **	0.63 **	0.74 **	0.73 **	0.74 **	0.64 **	0.67 **	0.76 **	1	
15. Other reasons	−0.07 **	−0.03	−0.01	−0.08 **	−0.09 **	0.69 **	0.68 **	0.65 **	0.70 **	0.69 **	0.63 **	0.59 **	0.66 **	0.65 **	1
M	1.53	1.90	1.85	9.18	8.75	47.06	37.90	34.80	50.55	39.04	23.29	27.26	23.52	39.32	27.18
SD	0.50	0.30	0.81	3.30	2.83	1.98	2.85	2.19	2.65	1.87	1.34	1.58	1.10	1.67	1.51

*p < 0.05, **p < 0.01.

### 3.3. The latent profile analysis of high school students’ sense of hope

In determining the number of latent categories, the model needs to be judged in conjunction with the fit indicators and the interpretability of the results. The optimal model is generally judged by the following indicators: (1) The smaller the values of AIC, BIC, and ABIC, the better the model fit (Muthén and Muthén, 2009). (2) A larger Entropy value indicates a better model fit. Entropy <0.60 corresponds to more than 20% of individuals with classification errors. Entropy ≥0.80 indicates a classification accuracy of more than 90%. Therefore, it is generally required to be >0.7 (Jung and Wickrama, 2008). (3) LMRT and BLRT are significant (*p* < 0.05), indicating that adding a profile can significantly improve the fit of the model (Nylund et al., 2007).

As can be seen from Table 2, the AIC, BIC, and ABIC of the model continue to decrease as the number of divided categories increases, and are lower and basically similar when divided into 3 and 4 categories. Meanwhile, the Entropy value tends to increase as the number of categories increases, and the Entropy value is higher between 0.92 and 0.94 when generating 2 and 3 categories, while the Entropy value is the highest when dividing into 2 categories. Moreover, the LMRT was not significant (*p* > 0.05) when divided into 4 categories, and the category probability was less than 5%. Therefore, combining the above model fit indicators, the type of high school students’ sense of hope status into 3 categories was optimal. Discriminant analysis was used to verify the accuracy of the results of the 4 categories of optimal model for the above potential profile analysis. It was found that the prediction accuracy of category 4 was slightly lower at 88.1%, while the prediction accuracy of the other three categories was higher at over 92%. This indicates that the optimal model results obtained from the selected latent profile analysis are more reasonable and credible.

**Table 2:** Latent profile model fit indicators for high school students’ sense of hope types.

Models	K	AIC	BIC	ABIC	Entropy	LMRT	BLRT	Category probability
1	16	32078.238	32163.387	32112.56				
2	25	26009.683	26142.73	26063.311	0.948	0.000	0.000	0.307/0.693
3	34	24675.381	24856.324	24748.315	0.924	0.000	0.000	0.243/0.263/0.494
4	43	24347.815	24576.655	24440.055	0.881	0.569	0.000	0.240/0.252/0.427/0.081

**Figure 1:**
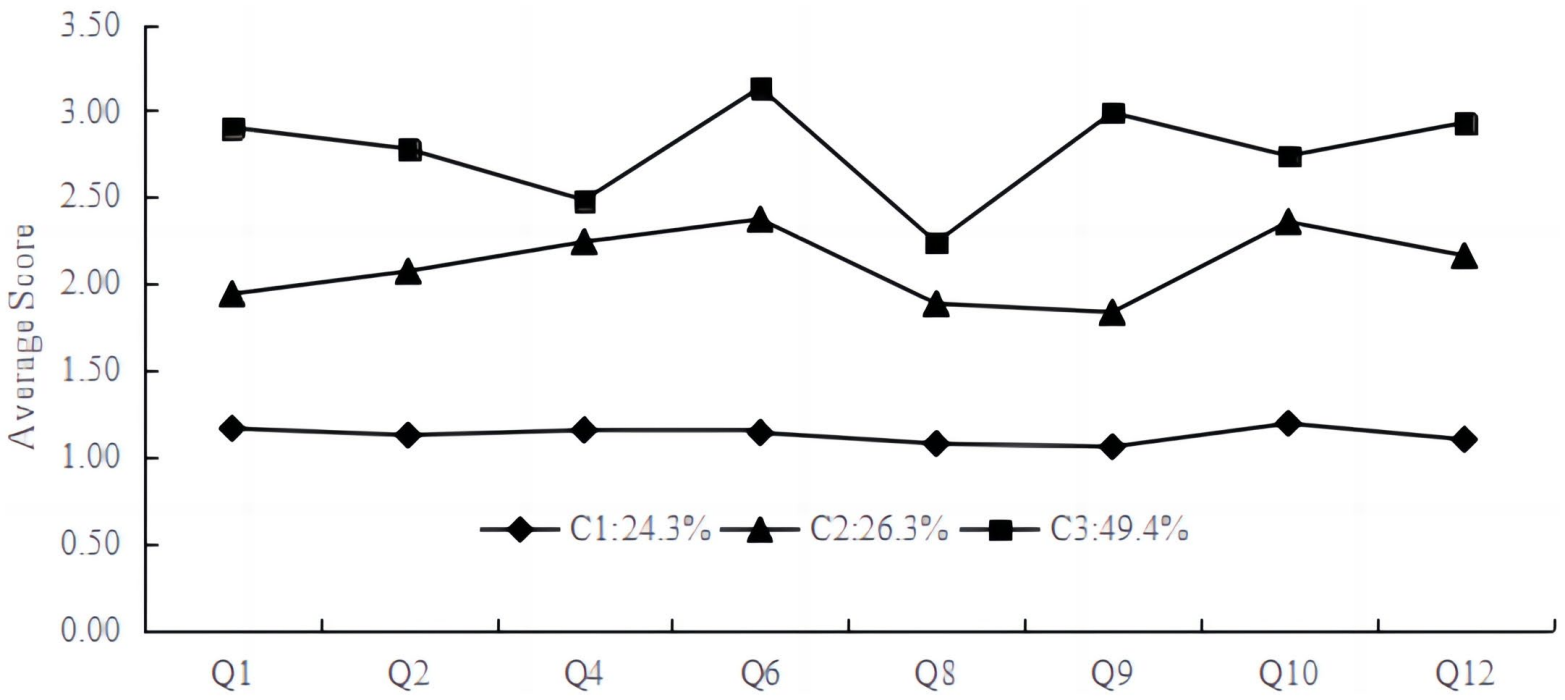
Average attribution probability of subjects in different latent categories. Pathway thinking (Q1, Q4, Q6, Q8), agency thinking (Q2, Q9, Q10, Q12).

As shown in Table 3, the average probability of belonging for high school students in each category ranged from 94 to 98%, which represents that the results of the three-category model are true and valid. The scores of the three potential categories on the sense of hope are shown in Figure 1. Category C1 scored significantly lower than C2 on each question, containing 24.26% of the subjects, and this profile was named the “negative sense of hope” group based on its score characteristics. Category C3 scored significantly higher than C2 on each question, containing 49.44% of the subjects, and this category was named the “positive sense of hope” group. The C2 category scored significantly higher than C1 on each question, but also significantly lower than C3, which included 26.30% of the subjects, and was named the “moderate sense of hope” group.

**Table 3:** Average attribution probability for subjects in different latent categories.

Categories	Number of subjects	Percentages	Attribution probability		
			C1	C2	C3
C1	367	24.26%	0.98	0.02	0.00
C2	398	26.30%	0.02	0.94	0.04
C3	748	49.44%	0.00	0.03	0.97

C1, negative sense of hope group; C2, moderate sense of hope group; C3, positive sense of hope group.

### 3.4. Differences in total scores and dimensions of sense of hope among high school students with different types of sense of hope

In order to explore whether there is heterogeneity in the classification of latent profiles of high school students’ sense of hope, we compared the three latent categories of high school students’ sense of hope profiles, and the results are shown in Table 4. The results showed that there were significant differences between the three types of high school students in their total sense of hope scores and in their scores on each dimension. Post-hoc multiple comparison analysis revealed that the two-way comparisons between the different types of high school students differed significantly on both the total hope sense score and its subscales. In other words, negative sense of hope high school students scored significantly lower on the two dimensions of sense of hope than moderate sense of hope high school students and positive sense of hope high school students. High school students with moderate sense of hope scored significantly higher on the two dimensions of sense of hope than high school students with a negative sense of hope and significantly lower than high school students with a positive sense of hope. This shows that the latent classification of high school students’ sense of hope can well distinguish the degree of high school students’ sense of hope, and it also shows that the latent classification is valid.

**Table 4:** Comparison of the status of sense of hope among high school students with different types of sense of hope.

	A latent profile of high school students’ sense of hope	
	Pathway thinking	Agency thinking
C1 (*N* = 367)	4.46 ± 0.84	4.92 ± 1.55
C2 (*N* = 398)	8.29 ± 1.21	8.49 ± 1.56
C3 (*N* = 748)	11.97 ± 1.32	10.76 ± 1.56
*F*-test	5045.65	1733.51
*η* ^2^	0.93	0.94
*Post hoc* test	C1 < C2 < C3	C1 < C2 < C3

C1, negative sense of hope group; C2, moderate sense of hope group; C3, positive sense of hope group.

### 3.5. Mental health of high school students with different types of sense of hope

The latent category of sense of hope was used as the independent variable, with all mental health indicators as dependent variables, and a one-way ANOVA test was performed. The results showed that the three groups of high school students differed significantly on all mental health indicators scores [*F* (*df* = 2) = 19.07, 51.87, 30.91, 33.07, 29.28, 24.16, 22.87, 22.80, 23.42, 25.04, η2 = 0.998, 0.994, 0.996, 0.997, 0.997, 0.996, 0.996, 0.998, 0.998, 0.997, *p* < 0.001]. Further two-way comparisons using difference tests revealed that high school students in the C3 (positive sense of hope) group scored significantly lower on all indicators of mental health than the rest of the groups. High school students in the C1 (negative sense of hope) group scored significantly higher on all indicators of mental health than the rest of the groups. The C2 (moderate sense of hope) group scored between the C3 and C1 groups on all indicators of mental health. In addition, somatization, depression, compulsive symptoms, and anxiety scores were significantly higher than the other factors, indicating that high school students have more serious internalizing problems in the emotional category, which is consistent with previous studies (Xia Fang and Yu, 2020). The descriptive data and the results of the test of variance for the three groups of subjects on the scores of mental health indicators are shown in Table 5.

**Table 5:** Descriptive data and test of variance (M ± SD) for the three groups of subjects on mental health.

	C1 (*N* = 367)	C2 (*N* = 398)	C3 (*N* = 748)	*F* (df = 2)	*η* ^2^	*Post hoc* test *Z*-value
Somatization	47.57 ± 1.05	46.72 ± 2.50	47.00 ± 1.96	18.78***	0.998	1 > 3 > 2
Compulsive symptoms	39.18 ± 1.48	37.49 ± 2.95	37.50 ± 3.11	50.07***	0.994	1 > 3 = 2
Interpersonal sensitivity	35.56 ± 1.12	34.46 ± 2.41	34.60 ± 2.37	28.03***	0.996	1 > 3 = 2
Depression	51.50 ± 1.30	50.11 ± 3.03	50.32 ± 2.82	30.14***	0.997	1 > 3 = 2
Anxiety	39.67 ± 0.91	38.79 ± 2.13	38.85 ± 2.00	28.45***	0.997	1 > 3 = 2
Hostility	23.70 ± 0.70	23.08 ± 1.59	23.20 ± 1.40	20.77***	0.996	1 > 3 = 2
Terror	27.73 ± 0.88	27.04 ± 1.76	27.15 ± 1.70	20.81***	0.996	1 > 3 = 2
Paranoia	23.84 ± 0.44	23.40 ± 1.30	23.43 ± 1.17	20.80***	0.998	1 > 3 = 2
Psychosis	39.83 ± 0.71	39.15 ± 2.01	39.15 ± 1.76	19.34***	0.998	1 > 3 = 2
Other reasons	27.65 ± 0.84	26.93 ± 1.74	27.09 ± 1.58	25.22***	0.997	1 > 3 = 2

C1, negative sense of hope group; C2, moderate sense of hope group; C3, positive sense of hope group. ***p < 0.001.

## 4. Discussion

In this study, a two-factor theoretical model was adopted to identify the types of high school students’ sense of hope using the latent profile analysis technique, and a total of three types of hope was identified, named positive sense of hope type, moderate sense of type, and negative sense of type. In addition, this study revealed the mental health of students with different types of sense of hope and found that high school students in the positive sense of hope group scored significantly lower on each of the SCL-90 indicators than the other two groups. In other words, high school students with a positive sense of hope possessed lower levels of negative psychology such as compulsive symptoms, interpersonal sensitivity, depression, anxiety, and hostility, and possessed better psychological status and physical condition.

### 4.1. The latent profile analysis of high school students’ sense of hope

In this study, latent profile analysis was used to explore the latent structure of high school students’ sense of hope based on the two-dimensional scores of their sense of hope, and the model with three latent profiles was selected as the optimal model based on a comprehensive consideration of relevant fitting indicators. The three profiles of the sense of hope do not differ qualitatively, but only quantitatively, that is, each profile has a consistent trend in the scores of each dimension. See Figure 1. This may reflect the fact that the sense of hope is a continuum from low to high at the individual level. In particular, the positive sense of group (C3) scored higher on all dimensions than the other profiles, with the highest mean score on the pathway thinking dimension, which accounted for 49.44% of the overall population, or about half of the population. The negative sense of hope group (C1) had lower scores on all dimensions than the other profiles and accounted for 24.26% of the total population, a small minority of the population. The moderate sense of group (C2) scored between the other two profiles in all dimensions and accounted for 26.30% of the population. While C2 and C3 together accounted for 75.74% of the overall, the degree of sense of hope in these two profile groups of high school students was moderate and above, which reflects that most high school students have a higher level of sense of hope. The reason for this phenomenon may be due to the current rapid economic and cultural development. Society promotes an educational philosophy that respects and supports students’ natural abilities. High school students are understood and supported by society, schools and families, and thereby have a stronger intrinsic motivation to pursue their goals and a higher sense of hope for the accomplishment of their tasks (Zhang et al., 2019).

### 4.2. Differences in total scores and dimensions of sense of hope among high school students with different types of sense of hope

The test of variance is shown in Table 4. High school students of the three sense of hope types differed significantly in their total sense of hope scores and in each dimension. High school students with a positive sense of hope scored significantly higher than high school students with moderate sense of hope and negative sense of hope on two dimensions of sense of hope, indicating that the latent classification of high school students’ sense of hope can well differentiate the degree of high school students’ sense of hope. In addition, both negative sense of hope high school students and moderate sense of hope high school students scored significantly lower on pathway thinking than on agency thinking. This suggests that high school students with sub-moderate sense of hope type lack self-efficacy to reach their goals and have difficulty in making connections between current and future thinking around goals to generate various methods or pathways to promote goal achievement (Zhu and Wu, 2019). High school students with a positive sense of hope scored higher in pathway thinking than in agency thinking. This may be due to the fact that when high school students experience frustration, they are more inclined to achieve maximum task completion by actively seeking new paths to mitigate the undesirable outcome resulting from the dilemma (Snyder, 2002). Specifically, students with positive sense of hope are able to form multiple implementation paths through divergent thinking patterns in the process of reaching their set goals, thus being able to positively adjust their mindset when experiencing adversity and to reach their goals in the most appropriate way (Liu, 2002).

### 4.3. Mental health of high school students with different types of sense of hope

This study used the BCH command to verify the different effects of different categories of sense of hope profiles on mental health. The results showed that the positive sense of hope profile was significantly higher than the other two profiles on all ten dimensions of negative mental health. The negative sense of hope profile scored the lowest, and the moderate sense of hope profile scored at an intermediate level. This is similar to the results of previous studies. For example, Snyder pointed out that compared to individuals with low levels of sense of hope, individuals with high levels of sense of hope would act in a positive way to explore new paths to achieve maximum goal accomplishment when faced with adversity, resulting in less individual ill feelings (Yanxiang, 2021). In terms of somatization, individuals with a high sense of hope have stronger immune systems, better resilience and regulation, and faster recovery in response to unexpected physical injuries and chronic illnesses (Yonghui, 2021). In terms of adverse emotions such as depression and anxiety, individuals with high levels of sense of hope have greater resilience, show less depression and anxiety, and have better behavioral tendencies than those with low levels of sense of hope (Yang et al., 2013). This was confirmed by Lewis and Kliewer in a study investigating children with sickle cell anemia, who concluded that sense of hope is closely linked to an individual’s self-esteem. Individuals with a high sense of hope have the emotional tendency to have high self-esteem and will reasonably control and mitigate negative emotions in the face of adversity, thereby reducing the production of undesirable behaviors.

The results of the study also found that the sense of hope score showed a negative relationship with the mental health score. These results confirm the prediction of this study that a sense of hope has a positive effect on the mental health of high school students. The positive correlation between sense of hope and psychological well-being has been confirmed by many studies. As a positive psychological energy, a sense of hope regulates the psychological state of individuals and motivates them to respond effectively to risky events, thus reducing the adverse consequences caused by risky events (Xiang Guangcan et al., 2020). Specifically, a higher sense of hope is conducive to enhancing individuals’ self-confidence and motivation in the face of adversity, improving their ability to control their emotions and behavioral performance, reducing adverse psychological states and emotional experiences, and thereby increasing their courage to face difficulties (Xia Fang and Yu, 2020). In a study of left-behind children, it was also found that a sense of hope could reduce negative emotions within individuals (O'Keefe and Wingate, 2013), increase their meaning in life and life satisfaction, and thus improve the problem behaviors of left-behind children (Zhang et al., 2022). In a study of a sense of hope intervention for stroke patients, it was found that a psychological intervention of sense of hope could improve limb motor function and neurological function by mobilizing patients’ subjective motivation and increasing their willingness to treat (Wang et al., 2022). Currently, there are more foreign studies related to sense of hope and somatization, showing that sense of hope has a positive effect on the development of somatization in individuals, however, related studies in China are less involved.

### 4.4. Education and intervention insights

This study examines the impact of two dimensions of sense of hope on mental health with high school students, which has implications for improving their mental health. Firstly, teachers should pay attention to the improvement of students’ pathway thinking and agency thinking in their daily learning. Previous research has shown that group counseling can increase students’ subjective motivation by guiding them to think about stated goals and stimulating individual intrinsic motivation in positive self-talk. This approach can deepen high school students’ deep understanding and positive attitudes toward goals, improving individuals’ pathway thinking and motivational awareness, and enhancing high school students’ emotional experience of sense of hope, which in turn enhances their hope traits (Yonghui, 2021). Therefore, teachers in high school should focus on students with negative and moderate sense of hope, especially the agency thinking of such students, and can intervene by improving their level of sense of hope through long-term counseling as well as by expanding new pathways.

Secondly, the educational field should actively increase the overall level of sense of hope in high school students to lay a solid psychological foundation for their future development. Some research findings suggest that increasing social support can help individuals experience less stress and have a greater sense of hope when faced with problems (Doolittle and Farrell, 2004). This shows that social support has a significant positive predictive effect on individuals’ sense of hope. Social support refers to the emotional and psychological experience of being respected, understood, and supported as perceived by the individual in society (Xiao Qian and Hua, 2013). It has been found that individuals with higher levels of family support will have stronger psychological motivation and emotional energy, and thus maintain higher levels of hope (Zhang et al., 2019). Therefore, families and schools should adopt supportive psychological attitudes and behavioral measures for high school students with a low sense of hope, so that high school students can perceive spiritual and emotional respect and support, and therefore have stronger motivation to pursue their set goals.

Finally, improving the mental health literacy of individuals is an important step in promoting the mental health of high school students. Mental health literacy should be integrated into the school curriculum according to the school’s own characteristics. And the curriculum design should be based on the law of physical and mental development of students in high school, and the content of mental health literacy training in high school should be reasonably selected to lay a solid institutional foundation for the normality, permanence and effectiveness of mental health literacy. This initiative is conducive to creating a positive general environment for mental health education, which in turn will improve the negative psychological conditions of high school students (Li and Chen, 2022).

### 4.5. Limitations and implications

The present findings also suggest some limitations and several directions for future research. Firstly, this study is a cross-sectional survey, which can only describe the current types of high school students’ sense of hope. The types of students’ sense of hope will change with time and environment, and future tracking studies can be adopted to focus on the changes in the types of high school students’ sense of hope. Secondly, the data collection of the variables in this study was all in the form of student self-reports, which inevitably have some deviation. A combination of other-rated and self-rated measures could be used in the future to obtain more realistic results. Finally, this study found that pathway thinking and agency thinking had a significant negative predictive effect on high school students’ negative emotions. This result seems to indicate that there is an inherent consistency between the two dimensions of sense of hope. Whether there is a difference in the prediction of positive affect between the two dimensions of sense of hope remains to be verified by more future research.

## 5. Conclusion

The central question of this study was the category characteristics of high school students’ sense of hope and its relationship with mental health. The results of the study fully validated the core questions of the study, and the specific findings are as follows. Firstly, sense of hope orientations in the high school student population can be classified into three types: positive sense of hope type, moderate sense of hope type, and negative sense of hope type. Among them, the moderate and positive hope types are more common in the group. Secondly, Positive sense of hope students have the lowest scores on SCL-90 factors such as somatization, compulsive symptoms, and interpersonal sensitivity. Negative Sense of Hope students scored the highest on the SCL-90 factors. Moderate sense of hope students scored in the middle of positive sense of hope and negative sense of hope.

This study is closely related to the field of knowledge. This study has an innovative contribution to the dimensionality of the division into category characteristics of sense of hope. In addition, this study further clarifies the correlation between the category characteristics of sense of hope and mental health. Meanwhile, the rationality of the two dimensions of sense of hope is validated to some extent in this study, that is, both agency thinking and pathway thinking have a significant negative predictive effect on negative emotions of high school students.

## Data availability statement

The raw data supporting the conclusions of this article will be made available by the authors, without undue reservation.

## Ethics statement

The studies involving human participants were reviewed and approved by First Affiliated Hospital, Shihezi University School of Medicine. Written informed consent to participate in this study was provided by the participants’ legal guardian/next of kin.

## Author contributions

RW and SD designed the experiment, prepared the manuscript, and made data analysis. YS collected data. YL corrected the whole language of the manuscript. CM gave technique supports and valuable suggestions in experiment designing. All authors contributed to the article and approved the submitted version.

## Funding

This research was supported by the Shihezi University Graduate Education Teaching Reform Research Project of China (2021Y-JGSJ11).

## Conflict of interest

The authors declare that the research was conducted in the absence of any commercial or financial relationships that could be construed as a potential conflict of interest.

## Publisher’s note

All claims expressed in this article are solely those of the authors and do not necessarily represent those of their affiliated organizations, or those of the publisher, the editors and the reviewers. Any product that may be evaluated in this article, or claim that may be made by its manufacturer, is not guaranteed or endorsed by the publisher.
